# (*E*)-5-hydroxy-7-methoxy-3-(2-hydroxybenzyl)-4-chromanone, a Major Homoisoflavonoid, Attenuates Free Fatty Acid-Induced Hepatic Steatosis by Activating AMPK and PPARα Pathways in HepG2 Cells

**DOI:** 10.3390/nu16203475

**Published:** 2024-10-14

**Authors:** Jae-Eun Park, Ji-Sook Han

**Affiliations:** 1 Department of Hotel Baking Technology, Busan Health University, Busan 49318, Republic of Korea; jepark@bhu.ac.kr; 2 Department of Food Science and Nutrition, Pusan National University, Busan 46241, Republic of Korea

**Keywords:** HMC, hepatic steatosis, AMPK, PPARα, HepG2

## Abstract

Background: (*E*)-5-hydroxy-7-methoxy-3-(2-hydroxybenzyl)-4-chromanone (HMC), a homoisoflavonoid isolated from *Portulaca oleracea*, has significant anti-adipogenesis potential; it regulates adipogenic transcription factors. However, whether HMC improves hepatic steatosis in hepatocytes remains vague. This study investigated whether HMC ameliorates hepatic steatosis in free fatty acid-treated human hepatocellular carcinoma (HepG2) cells, and if so, its mechanism of action was analyzed. Methods: Hepatic steatosis was induced by a free fatty acid mixture in HepG2 cells. Thereafter, different HMC concentrations (10, 30, and 50 µM) or fenofibrate (10 µM, a PPARα agonist, positive control) was treated in HepG2 cells.Results: HMC markedly decreased lipid accumulation and triglyceride content in free fatty acid-treated HepG2 cell; it (10 and 50 μM) markedly upregulated protein expressions of pAMP-activated protein kinase (AMPK) and acetyl-CoA carboxylase. HMC (10 and 50 μM) markedly inhibited the expression of sterol regulatory element-binding protein-1c, fatty acid synthase, and stearoyl-coA desaturase 1, which are the enzymes involved in lipid synthesis. Furthermore, HMC (10 and 50 μM) markedly upregulated the protein expression of peroxisome proliferator-activated receptor alpha (PPARα) and enhanced the protein expressions of carnitine palmitoyl transferase 1 and acyl-CoA oxidase 1. Conclusion: HMC inhibits lipid accumulation and promotes fatty acid oxidation by AMPK and PPARα pathways in free fatty acid-treated HepG2 cells, thereby attenuating hepatic steatosis.

## 1. Introduction

Hepatic lipid levels are generally balanced by the influx of free fatty acids (FFAs) and lipid synthesis and consumption. When the influx of FFAs and lipogenesis increases in the liver, excessive lipid accumulation occurs, leading to hepatic steatosis. Hepatic steatosis has long been considered a symptom of alcoholic liver disease; however, steatosis has been found even in the absence of alcohol consumption. As the number of obese or overweight individuals increases, nonalcoholic fatty liver disease has become a highly prevalent chronic liver disease. Nonalcoholic fatty liver disease includes nonalcoholic fatty liver (simple hepatic steatosis), inflammatory nonalcoholic steatohepatitis, advanced fibrosis, and liver cirrhosis [1]. Nonalcoholic fatty liver is an early, reversible stage of nonalcoholic fatty liver disease characterized by the accumulation of lipids without an inflammatory response [2]. Although the etiology of nonalcoholic fatty liver disease is not fully understood, it is believed that lipid metabolic disorders in the liver may play a crucial role in its initiation and progression [3]. Thus, the inhibition of lipid accumulation by regulating hepatic lipid metabolism is important for the treatment and prevention of fatty liver disease.

The major regulators of hepatic lipid metabolism that inhibit lipid accumulation are AMP-activated protein kinase (AMPK) and peroxisome proliferator-activated receptor alpha (PPARα) [4,5,6]. Acetyl-CoA carboxylase (ACC) and sterol regulatory element-binding protein 1c (SREBP-1c) are modulated by AMPK [7]. The activation of AMPK inhibits ACC and fatty acid synthase (FAS) by downregulating SREBP-1c and suppressing lipogenesis [8]. The PPARα, nuclear receptor protein activated by a ligand, modulates the expression of lipid oxidation transcription factors such as carnitine palmitoyl transferase 1 (CPT1) and acetyl-CoA oxidase 1 (ACOX1). Activated PPARα increases the expression of ACOX1 and CPT1 [9]. A reduction in PPARα expression was observed in individuals with hepatic steatosis, and a PPARα agonist was used to ameliorate this symptom [10,11]. Thus, AMPK and PPARα may be major targets for the prevention of hepatic steatosis.

The compound HMC ((*E*)-hydroxy-7-methoxy-3-(2′-hydroxybenzyl)-4-chromanone) was isolated from *P*. *oleracea*. It is homoisoflavonoid compound, a subclass of flavonoids with an additional carbon atom. Previous studies have revealed that HMC has antidiabetic, anti-obesity, and anti-inflammatory effects in cellular and diabetic animal model systems [12,13,14]. Although several effects and mechanisms of HMC in diseases have been elucidated, whether and how HMC improves nonalcoholic fatty liver in hepatocytes remains unclear. In this study, we investigated whether HMC could attenuate hepatic steatosis in FFA-stimulated human hepatocellular carcinoma (HepG2) cells. Furthermore, we determined the mechanism of action of the compound.

## 2. Material and Methods

### 2.1. Materials

**Figure 1:**
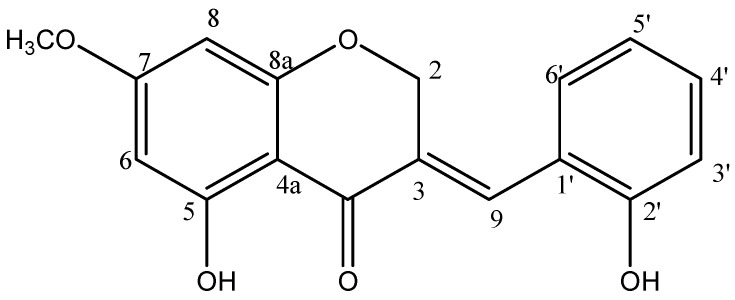
Chemical structure of HMC isolated from *P. oleracea*.

*P. oleracea* was collected from Hyosung-Food Inc. (Gangwon, Hongcheon, Republic of Korea). Extraction (using dichloromethane and methanol) and isolation of HMC from *P. oleracea* were performed using a previously established method in our laboratory [14]. HM-chromanone was isolated by reversed-phase HPLC (ODS-A, 75% aq. MeOH) using 5% MeOH in the CHCl_3_ fraction (0.35 g). The structure of the compound (Figure 1) was elucidated using a combination of spectroscopic methods, including HR/Mass and ¹H and ¹³C NMR.

### 2.2. Cell Culture

The HepG2 cells (human liver cancer cell line, immortal cells) were purchased from the Korean Cell Line Bank (KCLB No. 88065) and cultured in Dulbecco’s modified eagle’s medium (DMEM; HyClone Laboratories, Logan, UT, USA) at 37 °C in 5% CO_2_. The cells were then treated with a medium containing various concentrations of HMC and 1.0 mM FFA mixture (oleate/palmitate = 2:1) for 24 h. Fenofibrate (10 μM) served as the positive control, and compound C (10 µM) served as the AMPK inhibitor.

### 2.3. Treatment of Free Fatty Acid Mixture

The FFA mixture was prepared by conjugating oleate/palmitate (2:1) with fatty acid-free BSA [15]. Oleate and palmitate were dissolved in warmed BSA solution (45–50 °C) to give a stock of 8 mM FFA mixture. The final molar ratio of FFA mixture to BSA was 6:1.

### 2.4. Cell Viability

HepG2 cells were seeded a density of 1 × 10^4^ cells/well in 96-well plates and incubated at 37 °C in 5% CO_2_. Thereafter, different HMC concentrations (10, 30, and 50 µM) or fenofibrate (10 µM, a PPARα agonist, positive control) with the FFA (1.0 mM)-treated cells were incubated for 24 h. To each well, the MTT solution (Sigma, St. Louis, MO, USA) was added and incubated for 4 h. The absorbance was measured using the microplate reader (540 nm).

### 2.5. Oil Red O Staining

Cells were seeded at a density of 3 × 10^5^ cells/mL (1 mL) in 24-well plates for 24 h and then treated with the FFA mixtures. After 24 h of exposition, the HepG2 cells were washed with PBS and fixed in formalin for 2 h at room temperature. After washing with distilled H_2_O and once with 60% isopropanol, the fixed adipocytes were completely dried, stained with Oil Red O working solution for 1 h, and washed immediately with distilled H_2_O. Adipocytes were visualized using a microscope and image analysis. Intracellular triglyceride (TG) content was determined in cell lysates by an enzymatic colorimetric method and normalized by protein content, as previously described [16].

### 2.6. Western Blotting Analysis

The HepG2 cells were incubated with FFA stock solution (1.0 mM), followed by treatment with 10 and 50 μM HMC. The supernatants were quantified using Bio-Rad protein quantification reagent (Bio-Rad, Hercules, CA, USA). Samples were prepared by mixing buffer (Bio-Rad). The protein samples were separated by SDS-PAGE, and the acrylamide gel was transferred onto a nitrocellulose membrane. After blocking with TBS-T containing 5% skim milk for 1 h. The membrane was treated with the primary antibody (AMPK, PPARα, ACC, SREBP-1c, FAS and SCD-1, CPT1 and ACOX1) for 1 h. The secondary antibody (diluted 1:1000 with TBS-T) was incubated, and ECL solution was added to analyze protein expression in the dark using BioMax film. Relative protein expression was quantified by densitometric means using Multi Gauge v3.1.

### 2.7. Statistical Analysis

The results are expressed as mean ± standard deviation (SD, *n* = 3). Statistical analyses were performed using SPSS version 29.0 (IBM Corp., Armonk, NY, USA). The treatment groups were compared using one-way analysis of variance (ANOVA), followed by Tukey’s HSD test.

## 3. Results

### 3.1. Cell Viability with HMC

**Figure 2:**
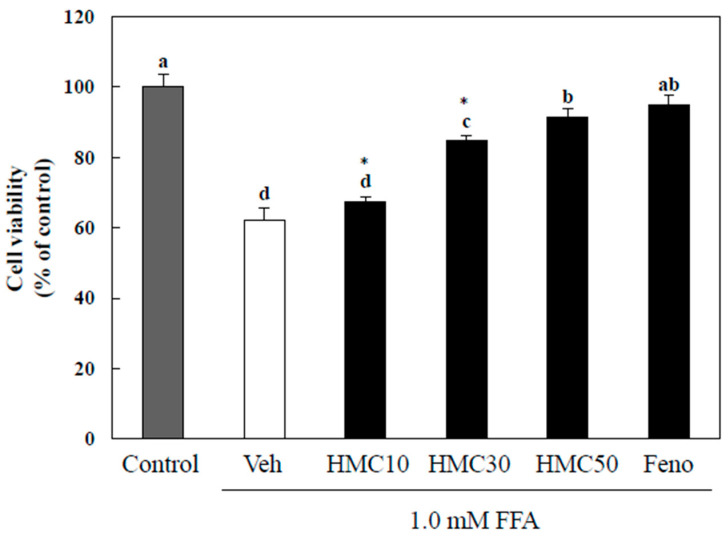
Cell Viability with HMC. Cell viability was stimulatied with 1.0 mM FFA stock solution (oleate/palmitate = 2:1) and then treatment with HMC (10, 30, and 50 μM) for 24 h. Each value is expressed as the mean ± standard deviation (*n* = 3), and values with different superscript letters were markedly different with *p* < 0.05 as analyzed using ANOVA, followed by Tukey’s HSD test. *: Statistically significant differences.

To test the cytotoxicity of the FFA and HMC, various HMC concentrations were incubated with the FFA in the cells (Figure 2). In HepG2 cells treated with an FFA, the cell viability was markedly lower compared to that in the normal control cells. However, the HMC treatment markedly increased the viability rate lowered by the FFA treatment.

### 3.2. HMC Inhibits Lipid Accumulation

**Figure 3:**
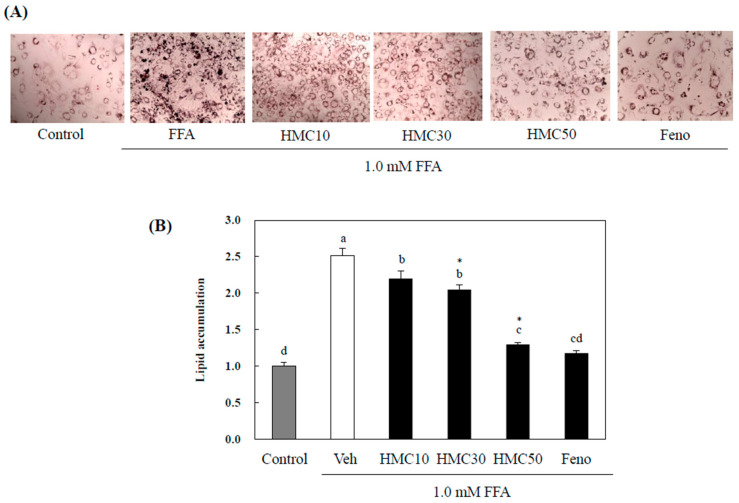
HMC inhibits lipid accumulation in FFA-induced hepatic steatosis. (**A**) Cells were fixed and stained with Oil Red O staining to visualize the lipid droplets by microscopy (magnification = 100X). Scale bar = 100 μM. (**B**) The effects of HMC on the inhibition of lipid accumulation. Each value is expressed as the mean ± standard deviation (*n* = 3), and values with different letters were markedly different with *p* < 0.05 as analyzed using ANOVA, followed by Tukey’s HSD test. *: Statistically significant differences.

Lipid accumulation is an important factor in fatty liver disease in hepatocytes. To investigate whether HMC could inhibit lipid accumulation in hepatocytes, HepG2 cells were treated with 10, 30, and 50 μM of HMC or 10 μM fenofibrate (positive control), and then exposed to a 1.0 mM FFA mixture (oleate/palmitate = 2:1) for 24 h. As shown in Figure 3, HMC markedly reduced lipid droplet formation in HepG2 cells exposed to FFAs. Lipid accumulation markedly increased by 2.55-fold upon exposure to FFA in HepG2 cells compared with that in the control. However, 10, 30, and 50 μM of HMC markedly reduced lipid accumulation by 2.20-, 2.03- and 1.34-fold, respectively, in HepG2 cells. Fenofibrate reduced lipid accumulation by 1.28% when used at a concentration of 10 μM. The 10 μM fenofibrate control and 50 μM HMC treatments showed similar values, with no significant difference.

### 3.3. HMC Reduces the Protein Expression of SREBP-1c, SCD-1, and FAS

**Figure 4:**
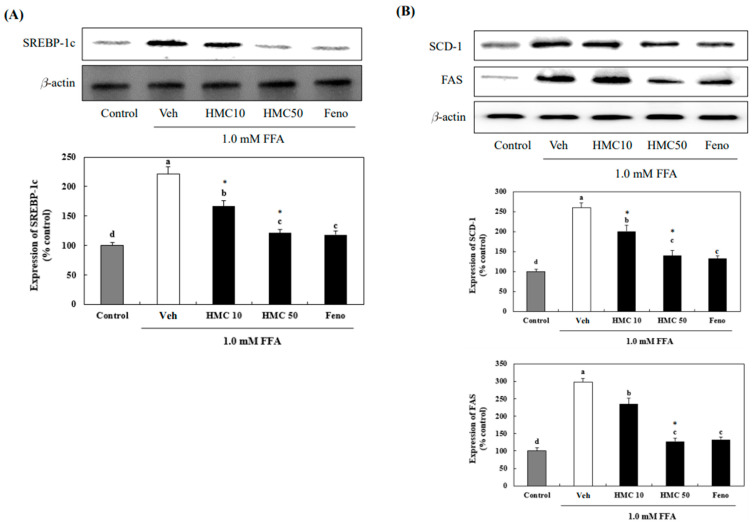
HMC inhibits the phosphorylation of SREBP-1c, SCD-1, and FAS in FFA-induced hepatic steatosis. (**A**) The protein expression of SREBP-1c. (**B**) The protein expression of SCD-1 and FAS. Each value is expressed as the mean ± standard deviation (*n* = 3), and values with different superscript letters were markedly different with *p* < 0.05 as analyzed using ANOVA, followed by Tukey’s HSD test. *: Statistically significant differences.

In FFA-exposed HepG2 cells, treatment with 10 and 50 μM HMC markedly reduced SREBP-1c expression by 166.43% and 121.11%, respectively, which was increased to 221.10% by FFAs (Figure 4). The 10 μM fenofibrate reduced SREBP-1c expression to 117.84%, which was not markedly different from the result of 50 μM HMC treatment. SCD-1 and FAS were also markedly increased to 259.98% and 297.45%, respectively, in HepG2 cells exposed to FFAs compared to that in the normal control cells. However, treatment with 10 and 50 μM HMC markedly decreased SCD-1 to 200.31% and 139.52% and FAS to 234.61% and 127.88%, respectively, compared to that in FFA-exposed HepG2 cells.

### 3.4. HMC Promotes AMPK and ACC in FFA-Exposed HepG2 Cells

**Figure 5:**
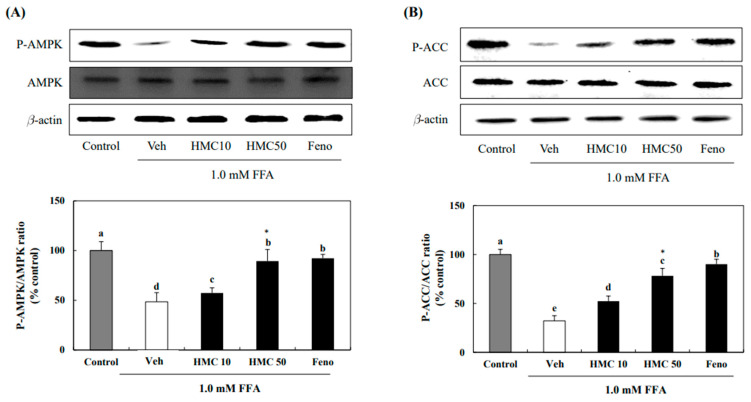
HMC promotes AMPK and ACC in FFA-induced hepatic steatosis. (**A**) The protein expression of AMPK. (**B**) The protein expression of ACC. Each value is expressed as the mean ± standard deviation (*n* = 3), and values with different superscript letters were markedly different with *p* < 0.05 as analyzed using one-way analysis of variance (ANOVA), followed by Tukey’s HSD test. *: Statistically significant differences.

To investigate whether HMC could induce AMPK in HepG2 cells exposed to FFA, HepG2 cells were treated with 10 and 50 μM of HMC and then exposed to FFAs. The AMPK phosphorylation markedly decreased by 48.51% in HepG2 cells exposed to FFAs compared to that in the control. However, treatment with 10 μM and 50 μM of HMC markedly increased the phosphorylation levels of AMPK to 57.39% and 89.38%, respectively (Figure 5). To confirm AMPK activation by HMC, ACC phosphorylation was investigated in HepG2 cells exposed to FFAs. The phosphorylation of ACC markedly decreased by 32.21% in HepG2 cells exposed to FFAs compared to that in the control. However, 10 μM and 50 μM of HMC markedly increased pACC to 52.30% and 78.19%, respectively.

### 3.5. HMC Inhibits the Expression of SREBP-1c, SCD-1, and FAS via Activating AMPK

**Figure 6:**
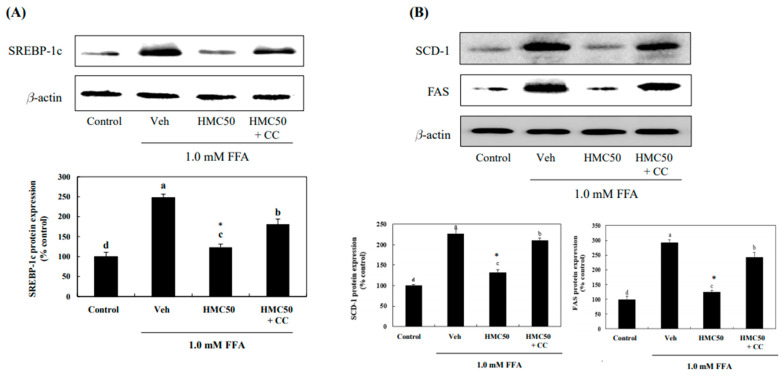
HMC inhibits the phosphorylation of SREBP-1c, SCD-1, and FAS via activating AMPK in FFA-induced hepatic steatosis. (**A**) The protein expression of SREBP-1c. (**B**) The protein expression of SCD-1 and FAS. Each value is expressed as the mean ± standard deviation (*n* = 3), and values with different superscript letters were markedly different with *p* < 0.05 as analyzed using one-way analysis of variance (ANOVA), followed by Tukey’s HSD test. *: Statistically significant differences.

To determine whether AMPK phosphorylation mediates the effects of HMC on the inhibition of lipogenesis-related transcription factors and enzymes, FFA-exposed HepG2 cells were treated with an AMPK inhibitor, compound C, in addition to HMC treatment. Exposure to FFAs increased SREBP-1c to 221.10% in HepG2 cells compared to that in the control; however, this increase was markedly inhibited by treatment with HMC (Figure 6). At a concentration of 50 μM, HMC markedly inhibited SREBP-1c expression by 123.43% in FFA-exposed HepG2 cells, but this effect was blocked by compound C. We also examined SCD-1, FAS, and SREBP-1c expression. Treatment with 50 μM HMC markedly decreased the expression of SCD-1 and FAS to 138.26% and 124.58%, respectively, compared with that in FFA-exposed HepG2 cells. However, these effects were blocked by compound C. When cells were treated with HMC combined with compound C, the expression of SCD-1 and FAS markedly increased. These results show that HMC may inhibit the expression of SREBP-1c, SCD-1, and FAS by activating AMPK in HepG2 cells exposed to FFA.

### 3.6. HMC Increases the Phosphorylation of PPARα, CPT1, and ACOX1

**Figure 7:**
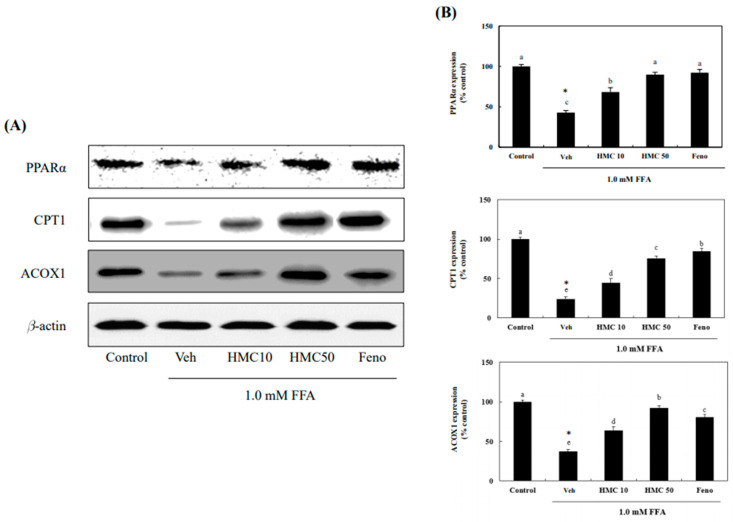
HMC increases the protein expression of PPARα, CPT1, and ACOX1. (**A**) The protein expression of PPARα, CPT1, and ACOX1. (**B**) Expression of PPARα, CPT1, and ACOX1. Each value is expressed as the mean ± standard deviation (*n* = 3), and values with different superscript letters were markedly different with *p* < 0.05 as analyzed using one-way analysis of variance (ANOVA), followed by Tukey’s HSD test. *: Statistically significant differences.

The HMC markedly increased the expression of PPARα, CPT1, and ACOX1 in FFA-exposed HepG2 cells. The expression level of PPARα was markedly reduced to 42.86% by FFAs in HepG2 cells compared to that in the control. However, the reduction was markedly recovered by treatment with 10 μM and 50 Μm of HMC to 68.37% and 89.84%, respectively (Figure 7). The PPARα expression level of the 10 μM fenofibrate control increased to 92.33%, and there was no significant difference between the 50 μM HMC treatment and 10 μM fenofibrate control. The expression levels of CPT1 and ACOX1, the critical fatty acid oxidation enzymes, were also markedly decreased to 23.97% and 37.38%, respectively, in FFA-exposed HepG2 cells compared to that in the control. However, treatment with 10 and 50 μM of HMC markedly increased the expression levels of CPT1 to 44.64% and 75.68% and ACOX1 to 63.75% and 92.33%, respectively. These results indicate that HMC could upregulate the expression of PPARα, CPT1, and ACOX1 in FFA-exposed HepG2 cells.

## 4. Discussion

Hepatic steatosis begins with lipid accumulation in the liver and is considered a major health problem. It could be caused by various factors, including the increased hepatic de novo lipogenesis, FFA uptake into the liver, and damaged fatty acid β-oxidation [17,18]. Insulin resistance also causes peripheral adipose lipolysis, which in turn promotes hepatic steatosis by inducing hepatic TG production when FFA supply to the liver is increased [19]. Patients with hepatic steatosis have approximately 60% of hepatic lipids induced from FFAs in adipose tissues. In contrast, healthy individuals obtain less than 5% of their hepatic lipids from de novo lipogenesis [20,21]. Thus, lipid accumulation due to excessive FFA influx and de novo lipogenesis is regarded as the main pathological factor in the development of hepatic steatosis. Reducing lipid accumulation by modulating hepatic lipid metabolism is an important strategy in the treatment of hepatic steatosis. We investigated whether HMC could reduce hepatic lipid accumulation, and if so, by what mechanism, in FFA-stimulated HepG2 cells.

Increased FFA influx results in hepatic lipid accumulation, leading to hepatic steatosis. Palmitic acid and oleic acid are common fatty acids. When used in vitro as a mixture, it can be an efficient model to investigate the effects of hepatic steatosis [22]. In this study, hepatic steatosis was induced using a FFA mixture (oleate/palmitate = 2:1) in HepG2 cells. The FFA mixture markedly increased lipid accumulation in HepG2 cell and HMC markedly decreased the lipid accumulation. Reduction in hepatic lipid accumulation has been associated with several lipid metabolism proteins [23,24]. Activation of AMPK inhibits lipogenesis, which utilizes ATP. Activated AMPK decreases SREBP-1c and inhibits the activation of ACC, SCD-1, and FAS [25]. Changes in the expression of these proteins related to hepatic lipogenesis were investigated to elucidate the mechanism by which HMC reduces hepatic lipid accumulation.

Expression of phosphorylated AMPK and ACC was markedly increased by HMC in FFA-exposed HepG2 cells. Lipogenesis is inhibited when AMPK is activated by phosphorylation. AMPK phosphorylates and inactivates the lipogenic enzyme ACC [26]. Therefore, HMC appears to be involved in the inhibition of hepatic lipid accumulation by phosphorylating AMPK and ACC. Homoisoflavone compounds markedly increase AMPK, and this activation was attributed to the C-3 methine group in the compounds [27]. Consequently, AMPK activation by HMC is possibly due to the C-3 methine group in its 16-carbon skeleton structure.

A major lipogenic transcription factor that is abundant in the liver is SREBP-1c [24]. Increased FFA influx into hepatocytes induces the overexpression of SREBP-1c, leading to de novo lipogenesis [28]. Activated SREBP-1c is translocated to the nucleus and upregulates the expression of lipogenic enzymes [29], which are the main enzymes involved in fatty acid and triglyceride synthesis in the liver, respectively. The synthesis of mono-unsaturated fatty acid from saturated fatty acetyl Co-A is catalyzed by SCD-1, and its deletion prevents the development of fatty liver [30,31]. The last step of fatty acid biosynthesis is catalyzed by FAS [32]. In this study, HMC markedly reduced the expression of SREBP-1c, SCD-1, and FAS in FFA-exposed HepG2 cells. However, it failed to reduce the expression of SREBP-1c, SCD-1, and FAS increased by FFAs in HepG2 cells pretreated with the AMPK inhibitor. This indicated that HMC reduced the expression of SREBP-1c, SCD-1, and FAS by activating AMPK. This was consistent with a study by Li et al. [25] where activated AMPK reduced the expression of SREBP-1c and subsequently inhibited SCD-1 and FAS activation [6].

Flavonoids can decrease lipid accumulation by increasing AMPK activation while suppressing SREBP-1c expression in HepG2 cells with palmitic acid [33]. Nobiletin, a major flavonoid in citrus fruit, reduced SREBP-1c and FAS via AMPK and inhibited hepatic lipid accumulation [34]. Hesperidin also inhibited lipid accumulation by increasing AMPK and decreasing SREBP-1c, ACC, and FAS expression in oleic acid-treated HepG2 cell [35]. Reports have indicated that AMPK activated by flavonoids could reduce lipogenesis by inhibiting SREBP-1c expression, thereby decreasing ACC and FAS expression.

The activation of PPARα promotes fatty acid β-oxidation and decreases the lipid content [36]. Agonists of PPARα have lipid-lowering action, such as lowering TGs, and they have long been used to treat dyslipidemia [37]. It markedly decreased triglyceride content in the liver of an insulin-resistant animal model [38]. The alleviation of fatty liver by atorvastatin, a lipid-regulating drug, has been demonstrated to be due to an increase in hepatic PPARα expression in rats [39]. The enzyme CPT1 controls acetyl CoA inflow and fatty acid β-oxidation in the mitochondrial membrane. It is a major regulator of fatty acid β-oxidation in the liver. An increase in its expression decreases hepatic triglyceride content and ameliorates fatty liver [40]. The rate-regulating enzyme, ACOX1, is the first enzyme involved in fatty acid β-oxidation [41]. Administration of a PPARα agonist enhances fatty acid β-oxidation enzymes, such as CPT1 and ACOX1, in rodent hepatocytes [42]. Thus, HMC markedly increased PPARα expression and enhanced CPT1 and ACOX1 expression in FFA-exposed HepG2 cells.

Quercetin enhanced PPARα and CPT-1 expression and decreased hepatic steatosis by increasing fatty acid oxidation [43]. Genistein, a major isoflavone, increased *CPT-1* and *ACOX1* through the activation of PPARα [44]. These reports indicate that compounds such as flavonoids and isoflavones could activate PPARα and increase the expression of genes involved in fatty acid catabolism. This study suggests that HMC could also stimulate β-oxidation through the PPARα activation and the increase in CPT1 and ACOX1 expression in hepatocytes.

## 5. Conclusions

**Figure 8:**
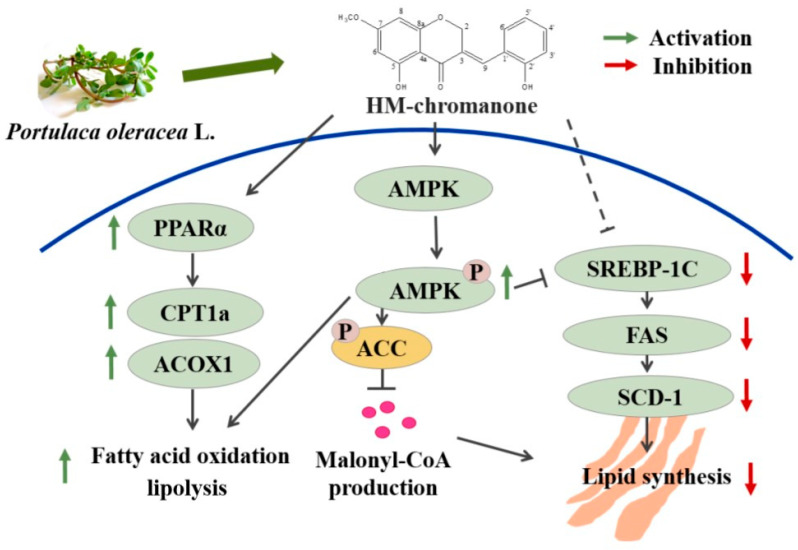
Proposed mechanism of attenuation of hepatic steatosis.

Our results demonstrate that HMC reduced lipid accumulation. This effect resulted from the activation of AMPK and PPARα signaling pathways, thereby suppressing SREBP-1c, FAS, and SCD-1 expression and increasing CPT1 and ACOX1 expression. Thus, lipogenesis was inhibited, and lipolysis was stimulated in the HepG2 cells (Figure 8).

## Data Availability

Data are available upon reasonable request to the corresponding author.
